# Incidence and influencing factors of subsyndromal delirium in elderly patients with pancreatic surgery: a prospective study

**DOI:** 10.3389/fpsyt.2025.1461707

**Published:** 2025-01-23

**Authors:** Hui-Qing Xu, Yun Wang, Ning-Ning Xia, Kuei-Ching Pan

**Affiliations:** ^1^ School of Nursing, Nanjing Medical University, Nanjing, Jiangsu, China; ^2^ The Affiliated BenQ Hospital of Nanjing Medical University, Nanjing, Jiangsu, China

**Keywords:** subsyndromal delirium, postoperative, risk factors, pancreas, elderly

## Abstract

**Objective:**

To prospectively investigatethe incidence and influencing factors of Subsyndromal delirium (SSD) in elderly patients undergoing pancreatic surgery.

**Methods:**

According to a prospective observational study, elderly patients (aged ≥60 years) who underwent pancreatic surgery in the pancreatic center of our hospital from August 2023 to February 2024 were selected. Patients were divided into SSD and Normal groups based on the evaluation of the Delirium Rating Scale-revised-98 in the first 1-4 days postoperatively. Multivariate logistic regression was performed to determine the influencing factors, and subject operating characteristic curves were used to assess the predictive effect of risk factors for subsyndromal delirium.

**Results:**

A total of 179 elderly pancreatic surgery patients were included in this study. 67 elderly patients developed subsyndromal delirium with an incidence of 37.43%. Multivariable Logistic regression revealed that risk factors for SSD included age, age-adjusted Charlson Comorbidity Index (aCCI), and postoperative fever, while and education level with senior high school or above was found to be protective factors. Receiver operating characteristic (*ROC*) curve showed that the combination of age and aCCI predicted SSD in elderly pancreatic surgery patients (Area Under Curve = 0.815, 95% Confidence Interval: 0.752 - 0.878), with sensitivity and specificity of 80.6% and 75.9%, respectively.

**Conclusion:**

The incidence of subsyndromal delirium after elderly pancreatic surgery was as high as 37.43%. Effective assessment and prevention of subsyndromal delirium are crucial. In the early postoperative period, special attention should be given to elderly patients with more preoperative comorbidities and lower education levels, and their temperature should be monitored in a timely manner.

## 1 Introduction

Delirium is the most common manifestation of brain dysfunction after major surgery. Current diagnostic criteria were proposed based on the Diagnostic and Statistical Manual of Mental Disorders, 5th edition (DSM-5), in which key diagnoses include acute onset and fluctuating course of symptoms, inattention, impaired level of consciousness, and cognitive disturbances (e.g., disorientation, memory impairment, and changes in language) (1). However, in clinical practice, some patients may exhibit symptoms that partially meet the diagnostic criteria for delirium but do not fully satisfy the criteria for delirium. This partial fulfillment of the diagnostic criteria for delirium is referred to as subsyndromal delirium (SSD). SSD is considered part of the severity spectrum of delirium (2). However, there is no established consensus on the definitions of SSD. SSD was first introduced by Levkoff in accordance with the Diagnostic and Statistical Manual of Mental Disorders (3). Until now, the diagnosis and evaluation criteria of SSD has been assessed with the help of delirium assessment tools. Intensive Care Delirium Screening Checklist (ICDSC) was introduced by Ouimet for SSD diagnosis (4), and then were used by Breu (5), Yamada (6) and Mailhot (7). Meagher used the Delirium Rating Scale-revised-98 (DRS-R-98) to diagnose SSD (8). Meanwhile, Gutierrez (9) and Tan (10) used the Confusion Assessment Method (CAM) to diagnose SSD. Similarly, Durlach (11) and Serafim (12) used the CAM -ICU. Diwell (13) used s-CAM. In addition, there were new symptoms after surgery when Li (14) and Denny (15) used CAM under additional criteria. Due to its insidious symptoms and low attention, it is prone to passive disposal due to delayed diagnosis. This is closely related to the adverse outcome of patients. Previous studies have suggested that delirium can compromise patient autonomy, increase mortality and dementia rates, reduce quality of life, and extend hospital stays (16). These effects have significant consequences for patients and their families (17), such as increased unplanned treatment and care costs, adding financial strain and exacerbating anxiety, and impact hospital efficiency and healthcare costs. These adverse outcomes emphasize the importance of early assessment and intervention for delirium. Empirical studies show that the probability of SSD progressing to delirium is 3.27 times higher than in normal patients (6), and the risk of death is 1.26 times higher than in normal patients (13). SSD can be prevented, and early assessment of the patients plays an important role in preventing the progression of SSD, as well as the transition to delirium. The prevalence of SSD varies considerably across different studies. The incidence of SSD varied widely among the various studies. Incidence rate of SSD in cardiac surgery and abdominal surgery were 34.2% to 37.8% (7, 14), and 11.7% to 36.7% (9, 18), respectively. However, up to 68% of elderly orthopedic patients have SSD (19), which is a common neuropsychiatric disorder in elderly patients.

As age increases, the degenerative changes in brain function and the decline in cognitive reserve make older patients more vulnerable to surgical stress (20). The brain’s nervous system is more susceptible to damage during this process, which increases the risk of postoperative delirium and SSD. Pancreatic surgery is particularly complex and high-risk, associated with complications such as infection, bleeding, and pancreatic leakage, making it of particular concern in older patients. Pancreatic surgery is typically divided into minimally invasive and open surgery. Despite the fact that minimally invasive surgery causes less tissue damage, its strict technical requirements and the possibility of converting to open surgery during the procedure have raised some controversy regarding its application in pancreatic surgery (21–23). In contrast, open surgery, with its more established technique, allows for better exposure of the lesion and surrounding tissues, and thus remains the standard approach (23). However, regardless of whether the surgery is minimally invasive or open, elderly patients often present with pancreatic metabolic abnormalities (24) and face numerous stressors during the perioperative period (25). These factors may exacerbate the physiological frailty of elderly patients and increase their vulnerability in pancreatic surgery, categorizing them as a high-risk group for SSD (26–28). Postoperative SSD is associated with many factors in the perioperative period (29), including advanced age, low education, poor cognitive status, and limb constraints, etc. There are few published studies on pancreatic surgery in the elderly given the wide variation in morbidity and factors affecting different types of surgery.

Here, a prospective observational study was performed to understand the incidence of SSD in elderly surgical patients in pancreatic centers and explore the influencing factors. This study provides a reference for the prevention of SSD and the perioperative management of the pancreas.

## 2 Materials and methods

### 2.1 Study design

This study was conducted in the pancreatic center of a teaching hospital in the eastern coastal areas of China. For the single-center, prospective cohort study design, the study protocol has been approved by the hospital ethics committee [NO. (2023) 583].

### 2.2 Inclusion and exclusion criteria

Inclusion criteria were as follows: (1) patients aged ≥60 years, regardless of gender; (2) patients with American Society of Anesthesiologists (ASA) classification of II-III; (3) patients postoperative entry Pancreatic center High Dependency Unit; and (4) patients undergoing partial or total pancreatic resection in the pancreatic center of our hospital.

Exclusion criteria included: (1) patients with visual, auditory, or language impairment, and inability to complete the assessment; and (2) patients with a preoperative history of psychiatric disorders.

### 2.3 Sample

Sample size was calculated according to the principle of survey study from 5 to 10 times of the variable. There were 13 survey scale independent variables in this study and sample size ranged from 65 to 130 cases. Considering 10% invalid data, 73 to 145 cases were needed.

The participants were patients who underwent elective surgery from August 2023 to February 2024 at our pancreatic center. After applying the criteria for inclusion and exclusion, a total of 206 patients were ultimately considered for this study. Written informed consent was obtained from all patients.

### 2.4 Measures

1. General data and Perioperative data questionnaire were developed after literature review and discussion by the research group. This involved in demographic data (age, gender, educational level), preoperative data [body mass index (BMI), drinking history, smoking history, disease history (hypertension, diabetes, cerebral infarction), surgery history, fasting time], intraoperative data (ASA Classification, blood loss, blood transfusion, operation time, anesthesia time), and postoperative fever [48 hours after> 38°C and returning to normal after 2~4 days is defined as postoperative fever (30)].

2. Age-adjusted Charlson Comorbidity Index (aCCI): It is a comprehensive index after Charlson proposes CCI scoring criteria including age, quantifying information on multiple comorbidities, and weighted age score, and has been widely used to reflect the overall functional status of patients (31). The final score for this scale was calculated based on weighted scores for 19 different diseases and different ages with a total score of 37. The higher the score, the more comorbidities and the worse the underlying status.

3. Nutrition Risk Screening 2002 (NRS-2002): Preferred tool for assessment of nutritional risk screening recommended by the European Society for Parenteral Nutrition in inpatients. It was first introduced in China in 2005 and applied to hospitalized patients by Chen Wei et al. (32). The scale included three aspects: disease severity score (0-3), nutritional status score (0-3), and age score (70 years plus 1 point). The total score ranged from 0 to 7, indicating patients at nutritional risk when the NRS-2002 score was 3 or above.

4. Fatigue, Resistance, Ambulation, Illness, Loss of weight (FRAIL): Proposed by the International Society for Nutrition and Aging in 2008, this component is based exclusively on patient self-reporting and is widely used for frailty screening in hospitalized patients (33). The scale includes five aspects: fatigue, resistance, decreased walking ability, five diseases, and weight loss of 5% greater than the original body weight within one year. The Chinese version of the frailty scale has a Cronbach’s alpha coefficient of 0.826. Each entry is scored as 0 (no) or 1 (yes) point, with a score range of 0 to 5. A score of 3 indicates frailty (34).

### 2.5 Assessment criteria for subsyndromal delirium

Delirium Rating Scale-revised-98 (DRS-R98), revised by Trzepacz et al. (35) in 1998, refined the cognitive function items based on the DRS scale and increased the evaluation items to 16, including 13 symptom severity scales and 3 additional diagnostic items. It can be used to identify other mental disorders, such as dementia and schizophrenia, and assess the severity of delusion. Items 1 to 13 include the non-cognitive partial scale (sleep-wake cycle, perceptual impairment, delusion, and thought process abnormalities) and the cognitive partial scale (attention, directional force, and short-term memory), and items 7 and 8 refer to the assessments of the activity types. The score of each item is 0 to 3 points, respectively. The internal consistency of the severity and total tables of DRS-R-98 was high with Cronbach α coefficients of 0.85 and 0.86, respectively (36). Patients was consider to have SSD at a DRS-R-98 score of 7-11 (8).

### 2.6 Data collection

All the data were collected and extracted by the members of the same research group (two researchers and one psychiatric expert). Both researchers had received unified training and guidance before the study to ensure the consistency of data measurement methods. After obtaining informed consent from the patients, preoperative surveys were conducted using the General and Perioperative Data Questionnaire, aCCI, NRS-2002, and FRAIL, and intraoperative data recording was improved within 24 hours after surgery. Considering that the SSD is a mild delirium, postoperative delirium mainly occurs within 24 to 72 hours after surgery, and assessment on four consecutive days detects delirium in 97% of cases (37). DRS-R-98 was used to assess postoperative patients with scores ranging from 7 to 11 on a scale of 7 to 11 every day at 6:00 p.m. for four consecutive days without diagnosis of delirium on that day being made, and was assessed for postoperative SSD. When the judgment of the two investigators was concordant, the corresponding results were recorded. If the two researchers’ judgments do not agree, they would seek a screening judgment from a psychiatrist.

### 2.7 Statistical analysis

Data were analyzed with SPSS 27.0. The normal distribution of numerical variables is expressed as mean ± standard deviation $\left(\bar{X}\;\pm \;S\right)$ , and the groups are compared by Student *t* test; The skewed distribution of numerical variables is presented as median and interquartile ranges [*M* (*P*_25_, *P*_75_)] and compared by Mann Whitney *U* test. Categorical variables were expressed as frequency numbers and compared by Chi-square test or Mann-Whitney *U* test between groups. The variables that showed statistically significant differences in the univariate analysis were included as independent variables, and multicollinearity testing was performed. The occurrence of SSD was used as the dependent variable, and binary logistic regression analysis was conducted to explore the independent factors influencing SSD. The results are reported as variance inflation factors (VIF), odds ratios (Odds Ratio, *OR*) and 95% confidence intervals (Confidence Interval, *CI*). The receiver operating characteristic (*ROC*) curve was used to assess the predictive efficacy of influencing factors for SSD. *P <*0.05 was considered to be statistically significant.

The variables that showed statistically significant differences in the univariate analysis were included as independent variables, and multicollinearity testing was performed.

The variables that showed statistically significant differences in the univariate analysis were included as independent variables, and multicollinearity testing was performed. The occurrence of SSD was used as the dependent variable. In addition, binary logistic regression analysis was conducted to explore the independent factors influencing SSD.

## 3 Results

### 3.1 The characteristics of the participants

**Figure 1:**
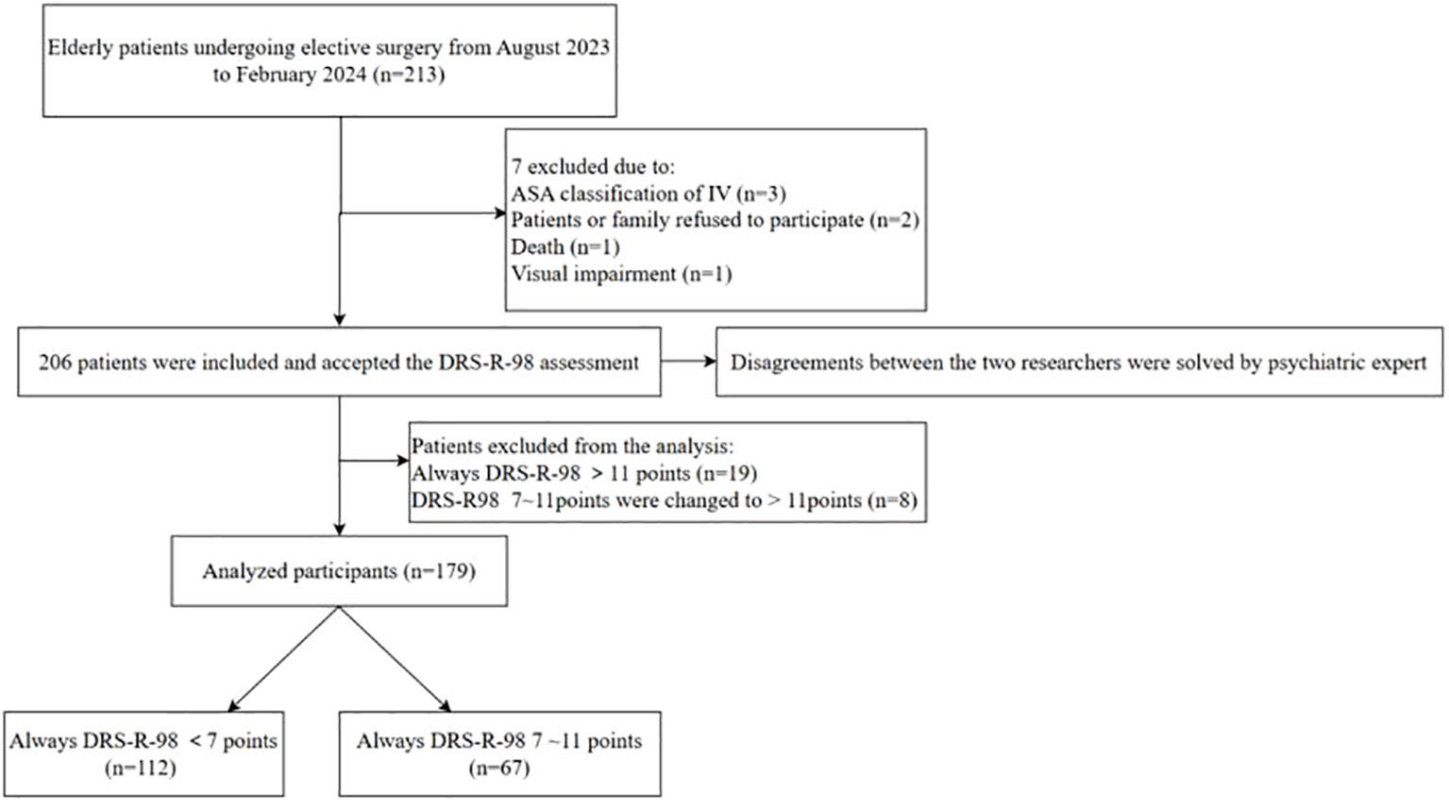
Flow chart of patients in study.

SSD was defined as a score between 7 and 11 on the DRS-R-98 and no diagnosis of delirium on the day of assessment. Any discrepancies in the assessment between the two researchers were resolved by consulting a psychiatric expert. A total of 206 elderly patients were evaluated. Patients with full delirium (n=19) and those who progressed into delirium (n=8) were excluded since this focused only on patients with SSD symptoms. Ultimately, 179 elderly pancreatic surgery patients were included (**Figure 1**). 67 elderly patients developed SSD after surgery, with an incidence of 37.43%. The mean age of the overall subjects was 69.04 ± 6.71 years, with 108 males and 71 females. In terms of education level, most of the subjects had a low education level, including 86 cases (48.04%) in primary school or below, 47 cases (26.27%) in junior middle school, 31 cases (17.32%) in high school and technical secondary school, and 15 cases (8.38%) in junior college or above. The ASA Classification in 58.66% (105/179) of the patients was at Grade II, the mean length of surgery was 3.68 ± 1.60 hours, and the mean maintenance time of anesthesia was 4.80 ± 1.71. **Table 1** shows the general and perioperative characteristics of the patients overall, as well as in both groups.

**Table 1:** General and perioperative characteristics of the patients.

Variables	Total N=179	Normal group (n=112)	SSD group (n=67)	Statistic	*P*-Value
Demographic characteristics					
Age (years $\bar{X}\pm S$ )	69.04 ± 6.71	66.37 ± 5.48	73.51 ± 6.22	-8.018^a^	<0.001*
Gender (n)				2.933^b^	0.087
Male	108	73	35		
Female	71	39	32		
Education (n)				-3.935^c^	<0.001*
Primary school and below	86	42	44		
Junior high school	47	32	15		
Senior high school/technical secondary school	31	25	6		
Junior college and above	15	13	2		
Preoperative characteristics					
BMI [kg/m^2^, (n)]				-1.806^c^	0.071
Normal (18.5~23.9)	106	62	44		
Thin (≤18.4)	16	9	7		
Overweight (24~27.9)	44	29	15		
Fat (≥28)	13	12	1		
Smoking history (n)	41	25	16	0.058^b^	0.810
Drinking history (n)	29	20	9	0.604^b^	0.437
aCCI (scores, $\bar{X}\pm S$ )	4.89 ± 1.131	4.48 ± 0.99	5.57 ± 1.02	-6.997^a^	<0.001*
ASA Classification (n)				10.439^b^	0.001*
II	105	76	29		
III	74	36	38		
History of cerebral infarction (n)	18	9	9	1.350^b^	0.245
History of diabetes (n)	52	26	26	4.945^b^	0.026*
History of hypertension (n)	89	54	35	0.272^b^	0.602
NRS-2002≥3 (n)	76	36	40	13.032^b^	<0.001*
History of surgery (n)	89	57	32	0.164^b^	0.685
Preoperative frailty (n)	40	18	22	6.790^b^	0.009*
Fasting time [h,M (P25, P75)]	10.00 (9.00,13.17)	9.92 (9.00,13.25)	10.67 (9.00,12.75)	-0.960^c^	0.337
Intraoperative characteristics					
Intraoperative blood loss [ml,M (P25, P75)]	150 (50,200)	150 (63,200)	150 (50,250)	-0.048^c^	0.961
Intraoperative blood transfusion (n)	20	9	11	2.968^b^	0.085
Duration of Surgery (hours, $\bar{X}\pm S$ )	3.68 ± 1.60	3.65 ± 1.46	3.73 ± 1.82	-0.334^a^	0.739
Duration of Anesthesia maintenance (hours, $\bar{X}\pm S$ )	4.80 ± 1.71	4.75 ± 1.53	4.88 ± 1.99	-0.483^a^	0.630
Postoperative characteristics					
Postoperative fever (°C, n)	38	9	29	31.146^b^	<0.001*

1. BMI, body mass index; ASA Classification, American Society of Anesthesiologists Classification; aCCI, age-adjusted Charlson Comorbidity Index; NRS-2002, Nutrition Risk Screening 2002; SSD, Subsyndromal delirium.

2. The presence of an asterisk (*) denotes statistically significant differences.

3. ^a^ *t*-test; ^b^Chi-square test; ^c^Mann–Whitney U-test.

### 3.2 Univariate analysis

The differences in age, education, history of diabetes, frailty, NRS - 2002 ≥ 3, aCCI, ASA classification, and postoperative fever between the SSD and Normal groups were statistically significant (*P <*0.05). No significant differences were found in other general characteristics and perioperative data (*P* > 0.05) (**Table 1**).

The variables showing statistically significant differences in the univariate analysis were included as independent variables, and multicollinearity testing was performed. It was clear that the variance inflation factors (VIF) were all below 5, indicating weak multicollinearity among the independent variables and low correlation between them. This confirms the suitability of the model for regression analysis (**Table 2**).

**Table 2:** Multicollinearity diagnosis results.

Variables	Tolerance	Variance Inflation Factor (VIF)
Age	0.443	2.256
Education	0.937	1.067
aCCI	0.352	2.845
ASA	0.717	1.396
History of diabetes	0.614	1.629
Preoperative frailty	0.688	1.453
NRS-2002≥3	0.782	1.279

ASA Classification, American Society of Anesthesiologists Classification; aCCI, age-adjusted Charlson Comorbidity Index; NRS-2002, Nutrition Risk Screening 2002.

### 3.3 Multivariable logistic regression analysis of SSD

A multivariate logistic stepwise regression model was constructed with the occurrence of SSD in the patient as the dependent variable (yes=1, no=0), and variables with statistically significant differences in univariate analyses, including age, aCCI, education, history of diabetes mellitus, ASA classification, NRS-2002 ≥3, preoperative frailty, and postoperative fever, as covariates. Numerical variables were entered with the original value, and categorical variables were assigned according to **Table 3**. The first one was selected as the reference category to screen the influencing factors of the occurrence of SSD in elderly pancreatic surgery patients (**Table 4**). The results showed that age (*OR* = 1.130, 95% *CI*=1.044~1.223, *P*=0.002), aCCI (*OR*=1.677, 95% *CI*=1.053~2.673, *P*=0.030) and Postoperative fever (*OR* = 5.437, 95% *CI*=2.027~14.581, *P*=0.001) were independent risk factors for SSD in elderly pancreatic surgery patients (*P <*0.05), while education level of high school/technical secondary school (*OR* = 0.180, 95% *CI*=0.051~0.637, *P*=0.008), and Junior college and above (*OR* = 0.121, 95% *CI*=0.018~0.814, *P*=0.030) were protective factors.

**Table 3:** Variable assignment method.

Variables	Assignment description
Gender	Male=1, Female=2
Education	primary school and below=1, Junior high school=2, Senior high school/technical secondary school=3, Junior college and above=4
Preoperative frailty	No=0, Yes=1
History of diabetes	No=0, Yes=1
ASA Classification	II=1, III=2
Postoperative fever	No=0, Yes=1

ASA Classification, American Society of Anesthesiologists Classification.

**Table 4:** Multivariable logistic regression analysis of impact factors of SSD.

Covariates	B	SE	Wald	*OR*	95% *CI*	*P*-Value
Age	0.144	0.041	12.158	1.155	1.065~1.253	<0.001
aCCI	0.521	0.239	4.739	1.684	1.053~2.692	0.029
Education	–	–	13.005	–	–	0.005
Education (1)	-0.75	0.475	2.493	0.472	0.186~1.199	0.114
Education (2)	-1.724	0.62	7.719	0.178	0.053~0.602	0.005
Education (3)	-2.749	1.023	7.213	0.064	0.009~0.476	0.007
Postoperative fever (1)	1.668	0.545	9.354	5.299	1.82~15.427	0.002

aCCI, age-adjusted Charlson Comorbidity Index; SE, standard error; OR, odds ratio; CI, Confidence Interval.

### 3.4 Predictive value of independent risk factors for SSD

**Figure 2:**
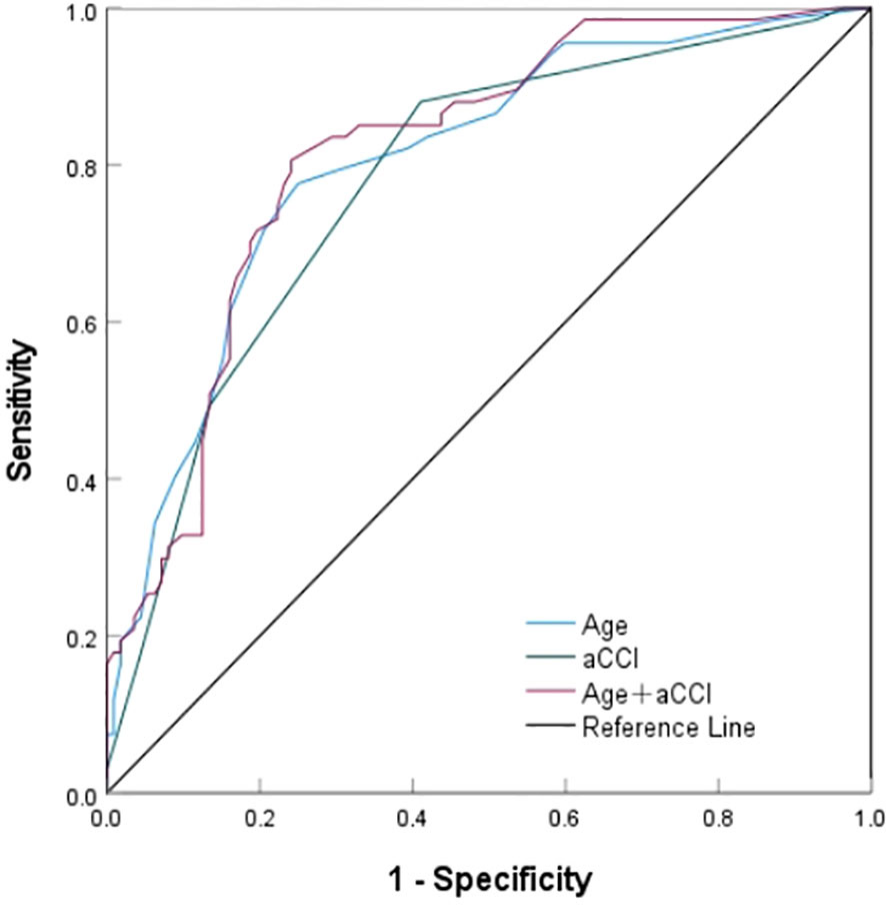
The ROC of risk factors to predict SSD.

Further, the value of screened independent risk factors in predicting SSD was evaluated (**Figure 2**, **Table 5**). The area under the *ROC* curve for age, aCCI, and a combination of both predict SSD was 0.804, 0.779, and 0.815, respectively, with sensitivities of 77.2%, 88.1%, 80.6%, and specificities of 75.0%, 58.9%, and 75.9%. According to the *ROC* curve analysis, the combination of age and aCCI was more appropriate for age, and the difference was statistically significant (*P <*0.001) when aCCI alone was used to predict SSD (0.815 [0.752-0.878] versus 0.804 [0.738-0.870] versus 0.779 [0.709-0.849]).

**Table 5:** The *ROC* parameters of risk factors to predict SSD.

Variables	The area under the curve (95% confidence interval)	*P*-Value	You-den index	Cut-off value	Cut-off value	
					Sensitivity	Specificity
Age	0.804(0.738~0.870)	<0.001	0.526	69.5	77.2%	75.0%
aCCI	0.779(0.709~0.849)	<0.001	0.470	4.5	88.1%	58.9%
Age+aCCI	0.815(0.752~0.878)	<0.001	0.565	–	80.6%	75.9%

aCCI, age-adjusted Charlson Comorbidity Index; *ROC*, receiver operating characteristic.

## 4 Discussion

### 4.1 Incidence of SSD in elderly patients with pancreatic surgery

The incidence of SSD was found to be 37.43%. This is a lower prevalence compared to the previously reported prevalence of more than two-thirds in the elderly after arthroplasty (19). There are two reasons for this difference. First, this was limited by the duration. Although SSD typically occurs in the early postoperative period, scholars often focus on the first three days after surgery because this time frame makes it easier to detect positive results and reduces interference from other postoperative factors, thereby saving resources and time. However, some patients may not exhibit symptoms for a week or more after surgery (38). Neglecting this part of the assessment may result in studies that do not adequately reflect the onset of delayed cognitive impairment symptoms. Future studies can extend the assessment duration to within one week postoperatively and conduct multicenter studies to gain a more comprehensive understanding of the mechanisms and influencing factors of SSD. Second, this study was a single-center investigation with some limitations in terms of sample representativeness. Since this study focuses on postoperative SSD in elderly patients, and given that poor physical condition and severe illness can negatively impact overall survival after surgery, doctors may prioritize older patients with relatively better underlying health to ensure the safety of the procedure. As a result, patients with poorer health or more comorbidities may not have been adequately considered. Postoperative cognitive impairment may manifest differently in this group of patients than in patients with better health. Additionally, sample size can be expanded in the future studies and a broader range of patient populations with different health conditions could be taken into account to enhance the generalizability and accuracy of the results. At the same time, more stringent patient selection criteria should be applied to avoid potential selection bias. It is noteworthy that most of the previously reported studies on pancreaticoduodenectomy rarely addressed SSD. Abnormal preoperative pancreatic metabolism and various perioperative stressor stimulation also make the increased vulnerability of elderly patients in pancreatic surgery become a high-risk group for SSD. However, the adverse consequences of SSD, such as cognitive impairment, prolonged hospitalization, and progression to delirium, necessitate early detection of SSD and targeted preventive measures in elderly patients undergoing pancreatic surgery.

### 4.2 Risk factors for SSD in elderly patients with pancreatic surgery

Risk factors for SSD include age, aCCI, and postoperative fever. However, as previous studies have found, age, comorbidities, and fever are also known risk factors for delirium, and these factors often play an important role in the patient’s recovery process. As age increases, older adults experience a gradual decline in neurologic and immune system function. Comorbidities further exacerbate the body’s stress response and affect cognitive function. Inflammation caused by fever can disrupt the balance of neurotransmitters, directly impacting brain function and increasing the likelihood of delirium (39). Therefore, age, comorbidities, and fever are risk factors for SSD and independent risk factors for the occurrence of delirium. It is crucial of understanding these factors to manage patients and prevent SSD and delirium. It has been reported that age is an independent risk factor for SSD (14, 29). Hwang et al. found a 3.85-fold (95% *CI*: 1.36 ~10.9295% *CI*: 1.36 ~10.92) increased risk of SSD in patients undergoing radical gastric surgery (18). With the increase of age, elderly patients have degenerative brain disease, decreased brain reserve function, and decreased physical tolerance to anesthesia and surgery (40). Besides, the nervous system is prone to secondary injury under acute stress during surgery. Elderly patients are at high risk for SSD due to the complexity, time-consuming nature, and high incidence of postoperative complications. Older patients are often considered at risk for cognitive impairment. Preoperative cognitive impairment has been shown to be a risk factor for SSD (29). However, this study did not fully consider the impact of preoperative cognitive status on patients. This stems from the fact that commonly used additional cognitive tests are cumbersome and require elderly patients to maintain high levels of focus and cooperation when responding to preoperative assessments, making it difficult to ensure their concentration and engagement during the tests. Hence, this variable was not thoroughly explored in this work. To address this limitation, more efforts be invested into the systematic assessment of preoperative cognitive status. For elderly patients undergoing preoperative evaluation, it may be beneficial to use more simplified and elderly-friendly cognitive assessment tools, such as the Mini-Mental State Examination (MMSE).

Consistent with previous meta-analyses (29), our evidence showed that patients with higher aCCI scores had an increased risk of developing SSD. This is associated with their overall functional status and comorbidities. The coexistence of chronic diseases can be attributed to the interaction between complex internal and external network systems after endogenous and exogenous stimulation, with chronic inflammation as the hub controlling the disease progression (41). Some scholars state that SSD belong to the prodromal stage or recovery stage, and the inflammation hypothesis is also a universally recognized mechanism. Accordingly, the coexistence of chronic diseases has been verified to be a risk factor for developing SSD in urological surgery (42) and cardiac surgery (14). Since prevention is far more effective than treatment, this study also found that the combined assessment of age and aCCI increases the clinical prediction of SSD.

Interestingly, consistent with the findings of Levkoff et al., postoperative fever is a major risk factor for developing SSD (3). Gao et al. found that patients with high temperature increased SSD 3.686 times (95% *CI*: 1.404 ~ 9.732) (43). Postoperative fever is related to the size of the surgery and the degree of body injury. Acute stress of surgery induces neuroendocrine system reaction, tissue damage to produce inflammation, inflammatory reaction increases the permeability of blood vessels, skin vasoconstriction, metabolic hyperactivity, and causes fever. Notably, a large accumulation of inflammatory factors can penetrate the blood-brain barrier leading to potential abnormal brain function in patients (44). In summary, in patients with various chronic diseases and postoperative fever, advance anti-inflammatory therapy can reduce the level of inflammation in the body, thus reducing the occurrence of SSD.

### 4.3 Protective factors for SSD in elderly patients with Pancreatic surgery

Notably, high school education and above is a protective factor for SSD in elderly pancreatic surgery patients, in agreement with Chen et al. (29) and Hwang et al. (18) studies. As confirmed in Bowman et al. (2), higher National Adult Reading Test (NART) scores are independently associated with resilience to delirium in orthopedic surgery patients, suggesting a protective effect. Lower literacy levels may be associated with lower cognitive reserve, which is thought to represent the ability of the patient’s brain to compensate for brain damage (45). Patients with lower educational levels are more likely to be affected by brain changes after surgery (46), leading to neuropsychiatric symptoms and SSD. By comparison, higher cognitive reserve in late life is associated with a lower incidence and severity of delirium in older adults undergoing surgery (47). Therefore, enhancing cognitive reserve through increased participation in cognitive activities is of great importance for reducing delirium, SSD, and other neuropsychiatric issues.

## 5 Conclusion

In brief, the incidence of SSD after elderly pancreatic surgery reached 37.43%. Effective assessment and prevention of subsyndromal delirium are crucial. The combined assessment of age and aCCI increased the early prediction of SSD, guiding attention to the screening work of high-risk patients before surgery. In the early postoperative period, special attention should be given to elderly patients with more preoperative comorbidities and lower education levels, and their temperature should be monitored in a timely manner. This helps to better understand the differences in clinical management and preventive approaches between delirium and SSD, and has significant implications for improving clinical care quality, optimizing postoperative preventive measures, and reducing the incidence of complications in patients.

There are several limitations in our study. Firstly, this study belongs to a single-center survey study, with some limitations in sample representativeness and possible selection bias. Secondly, we only assessed the occurrence of SSD within the first 4 days, overlooking patients with delayed onset. Finally, sufficient preoperative factors, especially the patients’ preoperative cognitive status, were not included. It should be noted that, as this study aims to focus solely on patients with SSD symptoms, patients with full delirium or those who progress to delirium were excluded from the analysis. Therefore, in future research, we plan to conduct a multi-center study, expand the sample size, extend the study period to one week postoperatively, and include more preoperative factors, especially cognitive function. We also plan to include individuals who develop delirium or progress from SSD to delirium in the analysis, with the goal of achieving a more comprehensive understanding.

## Data Availability

The original contributions presented in the study are included in the article/**Supplementary Material**. Further inquiries can be directed to the corresponding author.
