# Supplementary material for: Incidence and influencing factors of subsyndromal delirium in elderly patients with pancreatic surgery: a prospective study
Source: Front Psychiatry. 2025 Jan 23;16:1461707. doi: 10.3389/fpsyt.2025.1461707 (PMC11799266; doi:10.3389/fpsyt.2025.1461707)
Supplement: Supplementary file 1 [file DataSheet1.pdf]

Supplementary Material

1. Supplementary Figures and Tables

Table 1. General and perioperative characteristics of the patients

Variables	Total N=179	Normal group（n=112）	SSD group（n=67）	Statistic	*P*-Value
**Demographic characteristics**					
Age（years，$\bar{X}$ ±S）	69.04±6.71	66.37±5.48	73.51±6.22	-8.018[a]	＜0.001*
Gender（n）				2.933[b]	0.087
Male	108	73	35		
Female	71	39	32		
Education（n）				-3.935[c]	＜0.001*
Primary school and below	86	42	44		
Junior high school	47	32	15		
Senior high school/ technical secondary school	31	25	6		
Junior college and above	15	13	2		
**Preoperative characteristics**					
BMI [kg/m^2,（n）]				-1.806[c]	0.071
Normal（18.5~23.9）	106	62	44		
Thin （≤18.4）	16	9	7		
Overweight（24~27.9）	44	29	15		
Fat（≥28）	13	12	1		
Smoking history（n）	41	25	16	0.058[b]	0.810
Drinking history（n）	29	20	9	0.604[b]	0.437
aCCI（scores，$\bar{X}$ ±S）	4.89±1.131	4.48±0.99	5.57±1.02	-6.997[a]	＜0.001*
ASA Classification（n）				10.439[b]	0.001*
II	105	76	29		
III	74	36	38		
History of cerebral infarction（n）	18	9	9	1.350[b]	0.245
History of diabetes（n）	52	26	26	4.945[b]	0.026*
History of hypertension（n）	89	54	35	0.272[b]	0.602
NRS-2002≥3（n）	76	36	40	13.032[b]	＜0.001*
History of surgery（n）	89	57	32	0.164[b]	0.685
Preoperative frailty（n）	40	18	22	6.790[b]	0.009*
Fasting time [h,M(P25, P75)]	10.00（9.00,13.17）	9.92（9.00,13.25）	10.67（9.00,12.75）	-0.960[c]	0.337
**Intraoperative characteristics**					
Intraoperative blood loss [ml,M(P25, P75)]	150（50,200）	150（63,200）	150（50,250）	-0.048[c]	0.961
Intraoperative blood transfusion（n）	20	9	11	2.968[b]	0.085
Duration of Surgery （hours, $\bar{X}$ ±S）	3.68±1.60	3.65±1.46	3.73±1.82	-0.334[a]	0.739
Duration of Anesthesia maintenance（hours，$\bar{X}$ ±S）	4.80±1.71	4.75±1.53	4.88±1.99	-0.483[a]	0.630
**Postoperative characteristics**					
Postoperative fever（℃，n）	38	9	29	31.146[b]	＜0.001*

1. Abbreviations: BMI, body mass index; ASA Classification, American Society of Anesthesiologists Classification; aCCI, age-adjusted Charlson Comorbidity Index; NRS-2002, Nutrition Risk Screening 2002; SSD, Subsyndromal delirium.

2. The presence of an asterisk (*) denotes statistically significant differences.

3. a *t*-test; b Chi-square test; c Mann–Whitney U-test.

Table 2. Multicollinearity Diagnosis Results

Variables	Tolerance	Variance Inflation Factor (VIF)
Age	0.443	2.256
Education	0.937	1.067
aCCI	0.352	2.845
ASA	0.717	1.396
History of diabetes	0.614	1.629
Preoperative frailty	0.688	1.453
NRS-2002≥3	0.782	1.279

Abbreviations: ASA Classification, American Society of Anesthesiologists Classification; aCCI, age-adjusted Charlson Comorbidity Index; NRS-2002, Nutrition Risk Screening 2002;

Table 3. Variable assignment method

Variables	Assignment description
Gender	Male=1, Female=2
Education	primary school and below=1, Junior high school=2, Senior high school/ technical secondary school=3, Junior college and above=4
Preoperative frailty	No=0, Yes=1
History of diabetes	No=0, Yes=1
ASA Classification	II=1, III=2
Postoperative fever	No=0, Yes=1

Abbreviations: ASA Classification, American Society of Anesthesiologists Classification;

Table 4. Multivariable logistic regression analysis of impact factors of SSD

Covariates	B	SE	Wald	*OR*	95% *CI*	*P*-Value
Age	0.144	0.041	12.158	1.155	1.065~1.253	＜0.001
aCCI	0.521	0.239	4.739	1.684	1.053~2.692	0.029
Education	-	-	13.005	-	-	0.005
Education（1）	-0.75	0.475	2.493	0.472	0.186~1.199	0.114
Education（2）	-1.724	0.62	7.719	0.178	0.053~0.602	0.005
Education（3）	-2.749	1.023	7.213	0.064	0.009~0.476	0.007
Postoperative fever（1）	1.668	0.545	9.354	5.299	1.82~15.427	0.002

Abbreviations: aCCI, age-adjusted Charlson Comorbidity Index; SE, standard error; OR, odds ratio; CI, Confidence Interval.

Table 5. The *ROC* parameters of risk factors to predict SSD

Variables	The area under the curve (95% confidence interval)	*P*-Value	You-den index	Cut-off value	Cut-off value	
					Sensitivity	Specificity
Age	0.804（0.738~0.870）	＜0.001	0.526	69.5	77.2%	75.0%
aCCI	0.779（0.709~0.849）	＜0.001	0.470	4.5	88.1%	58.9%
Age＋aCCI	0.815（0.752~0.878）	＜0.001	0.565	-	80.6%	75.9%

Abbreviations: aCCI, age-adjusted Charlson Comorbidity Index; receiver operating characteristic (*ROC*);

2. Supplementary Figures

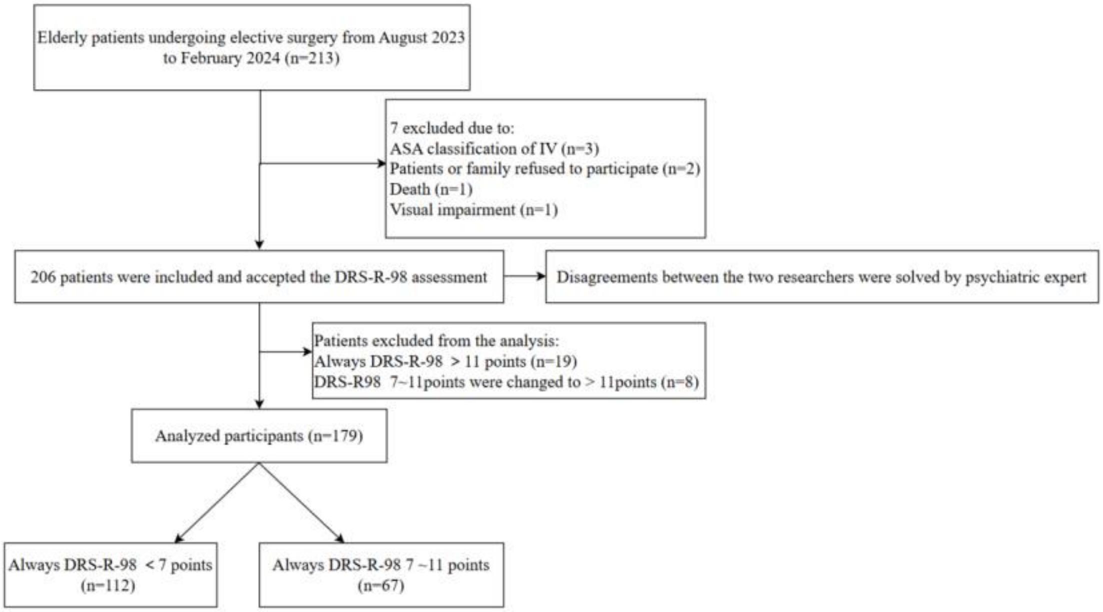

Figure 1. Flow chart of patients in study.

ASA, American Society of Anesthesiologists; DRS-R-98, The Delirium Rating Scale-revised-98.

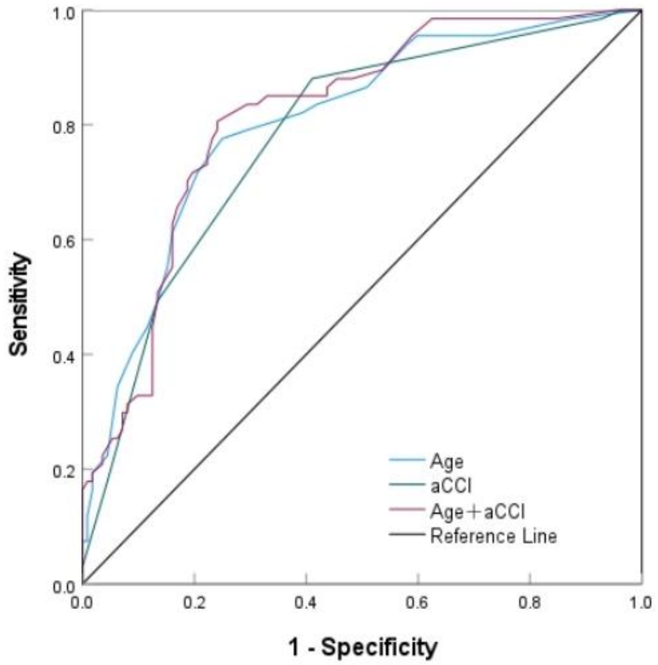

Figure 2. The *ROC* of risk factors to predict SSD

3. Supplementary Scale

General data and Perioperative data questionnaire

**General data**	Age:______years	Gender: Male Female
	Educational level:<br>Primary school and below; Junior high school;<br>Senior high school/ technical secondary school; Junior college and above;	
**Perioperative data**		
Preoperative data	Body mass index:<br>Normal (18.5~23.9kg/m^2) Thin (≤18.4kg/m^2) Overweight (24~27.9kg/m^2) Fat (≥28kg/m^2)	
	Drinking history: Yes No	Smoking history: Yes No
	Disease history: Hypertension; Diabetes; Cerebral infarction;	
	Surgery history: Yes No	Fasting time:
Intraoperative data	ASA Classification: II III	
	Blood loss:_______(ml)	Blood transfusion: Yes No
	Operation time:_______(h)	Anesthesia time:_______(h)
Postoperative data	Postoperative fever: Yes No	

age-adjusted Charlson Comorbidity Index

Score	Comorbidity
1	Ages 50-59, Myocardial infarction, Congestive heart failure, Cerebral vascular disease, Dementia, Chronic obstructive pulmonary disease, Connective tissue disease, Ulcer disease, Mild liver disease, Diabetes
2	Ages 60-69, Hemiplegia, Moderate/severe renal disease, Diabetes with end-organ damage, Any tumor, Leukemia, Lymphoma
3	Ages 70-79, Moderate/severe liver disease
4	Ages 80 and above
6	Metastatic solid tumor, AIDS

Nutrition Risk Screening 2002

Table 1 Initial screening

1	Is BMI <20.5?	Yes	No
2	Has the patient lost weight within the last 3 months?		
3	Has the patient had a reduced dietary intake in the last week?		
4	Is the patient severely ill?（e.g. in intensive therapy）		

Yes: If the answer is 'Yes' to any question, the screening in Table 3.2 is performed

No: If the answer is 'No' to all questions, the patient is re-screened at weekly intervals. If the patient e.g. is scheduled for a major operation, a preventive nutritional care plan is considered to avoid the associated risk status.

Table 2 Final screening

	Impaired nutritional status		Severity of disease (≈ increase in requirements)
Absent Score 0	Normal nutritional status	Absent Score 0	Normal nutritional requirements
Mild Score 1	Weight loss >5% in 3 months or Food intake below 50-75% of normal requirement in preceding week	Mild Score 1	Hip fracture* Chronic patients, in particular with acute complications, cirrhosis*, COPD*, Chronic hemodialysis, diabetes, oncology
Moderate Score 2	Weight loss >5% in 2 months or BMI 18.5-20.5 + impaired general condition or Food intake 25-60% of normal requirement in preceding week	Moderate Score 2	Major abdominal surgery*, Stroke, Severe pneumonia, hematologic malignancy
Severe Score 3	Weight loss >5% in l month (>15% in 3 months) or BMI <l8.5 +impaired general condition or Food intake 0-25% of normal requirement in preceding week	Severe Score 3	Head injury*, Bone marrow transplantation*, Intensive care patients (APACHE >10)
Impaired nutritional status Score: _____+ Severity of disease Score:_____= Total score:			
Age	if ≥70 years：add 1 to total score above		=age-adjusted total score

Fatigue, Resistance, Ambulation, Illness, Loss of weight

Item	Yes/No
1. Fatigue: Are you fatigued?	
2. Resistance: Cannot walk up 1 flight of stairs?	
3. Aerobic: Cannot walk 1block?	
4. Illnesses: Do you have more than 5 illnesses?	
5. Loss of weight: Have you lost more than 5% of you weight in the past 6 months?	

The Delirium Rating Scale-revised-98

Item	Severity Scale
Sleep–wake cycle	0. Not present<br>1. Mild sleep continuity disturbance at night or occasional drowsiness during the day<br>2. Moderate disorganization of sleep-wake cycle (e.g., falling asleep during conversations, napping during the day or several<br>brief awakenings during the night with confusion/behavioral changes or very little nighttime sleep)<br>3. Severe disruption of sleep-wake cycle (e.g., day-night reversal of sleep-wake cycle or severe circadian fragmentation with<br>multiple periods of sleep and wakefulness or severe sleeplessness.)
Perceptual disturbances	0. Not present<br>1. Mild perceptual disturbances (e.g., feelings of derealization or depersonalization; or patient may not be able to

	discriminate dreams from reality)<br>2. Illusions present<br>3. Hallucinations present
Delusion	0. Not present<br>1. Mildly suspicious, hypervigilant, or preoccupied<br>2. Unusual or overvalued ideation that does not reach delusional proportions or could be plausible<br>3. Delusional
Affective lability	0. Not present<br>1. Affect somewhat altered or incongruent to situation; changes over the course of hours; emotions are mostly under self-control<br>2. Affect is often inappropriate to the situation and intermittently changes over the course of minutes; emotions are not<br>consistently under self-control, though they respond to redirection by others<br>3. Severe and consistent disinhibition of emotions; affect changes rapidly, is inappropriate to context, and does not respond<br>to redirection by others
Language	0. Normal language<br>1. Mild impairment including word-finding difficulty or problems with naming or fluency<br>2. Moderate impairment including comprehension difficulties or deficits in meaningful communication (semantic content)<br>3. Severe impairment including nonsensical semantic content, word salad, muteness, or severely reduced comprehension
Thought process	0. Normal thought processes<br>1. Tangential or circumstantial<br>2. Associations loosely connected occasionally, but largely comprehensible<br>3. Associations loosely connected most of the time
Motor agitation	0. No restlessness or agitation<br>1. Mild restlessness of gross motor movements or mild fidgetiness<br>2. Moderate motor agitation including dramatic movements of the extremities, pacing, fidgeting, removing intravenous lines,<br>etc.<br>3. Severe motor agitation, such as combativeness or a need for restraints or seclusion
Motor retardation	0. No slowness of voluntary movements<br>1. Mildly reduced frequency, spontaneity or speed of motor movements, to the degree that may interfere somewhat with the<br>assessment<br>2. Moderately reduced frequency, spontaneity or speed of motor movements to the degree that it interferes with participation in activities or self-care<br>3. Severe motor retardation with few spontaneous movements
Orientation	0. Oriented to person, place and time<br>1. Disoriented to time (e.g., by more than 2 days or wrong month or wrong year) or to place (e.g., name of building, city,

	state), but not both<br>2. Disoriented to time and place<br>3. Disoriented to person
Attention	0. Alert and attentive<br>1. Mildly distractible or mild difficulty sustaining attention, but able to refocus with cueing. On formal testing makes only minor errors and is not significantly slow in responses<br>2. Moderate inattention with difficulty focusing and sustaining attention. On formal testing, makes numerous errors and either requires prodding to focus or finish the task<br>3. Severe difficulty focusing and/or sustaining attention, with many incorrect or incomplete responses or inability to follow instructions. Distractible by other noises or events in the environment
Short-term memory	0. Short-term memory intact<br>1. Recalls 2/3 items; may be able to recall third item after category cueing<br>2. Recalls 1/3 items; may be able to recall other items after category cueing<br>3. Recalls 0/3 items
Long-term memory	0. No significant long-term memory deficits<br>1. Recalls 2/3 items and/or has minor difficulty recalling details of other long-term information<br>2. Recalls 1/3 items and/or has moderate difficulty recalling other long-term information<br>3. Recalls 0/3 items and/or has severe difficulty recalling other long-term information
Visuospatial ability	0. No impairment<br>1. Mild impairment such that overall design and most details or pieces are correct; and/or little difficulty navigating in his/her surroundings<br>2. Moderate impairment with distorted appreciation of overall design and/or several errors of details or pieces; and/or needing repeated redirection to keep from getting lost in a newer environment despite, trouble locating familiar objects in immediate environment<br>3. Severe impairment on formal testing; and/or repeated wandering or getting lost in environment
Item	Optional Diagnostic Items
Temporal onset	0. No significant change from usual or longstanding baseline behavior<br>1. Gradual onset of symptoms, occurring over a period of several weeks to a month<br>2. Acute change in behavior or personality occurring over days to a week<br>3. Abrupt change in behavior occurring over a period of several hours to a day
Fluctuation	0. No symptom fluctuation<br>1. Symptom intensity fluctuates in severity over hours<br>2. Symptom intensity fluctuates in severity over minutes
Physical disorder	0. None present or active<br>1. Presence of any physical disorder that might affect mental state<br>2. Drug, infection, metabolic disorder, CNS lesion or other medical problem that specifically can be implicated in causing the altered behavior or mental state
